# A Fuzzy Logic Approach for the Reduction of Mesh-Induced Error in CFD Analysis: A Case Study of an Impinging Jet

**DOI:** 10.3390/e21111047

**Published:** 2019-10-27

**Authors:** Marcin Sosnowski, Jaroslaw Krzywanski, Radomir Scurek

**Affiliations:** 1 Faculty of Science and Technology, Jan Dlugosz University in Czestochowa, Armii Krajowej 13/15, 42-200 Czestochowa, Poland; j.krzywanski@ujd.edu.pl; 2 Faculty of Safety Engineering, Technical University of Ostrava, Lumirova 630/13, 700 30 Ostrava-Vyskovice, Czech Republic; radomir.scurek@vsb.cz

**Keywords:** impinging jet, domain discretization, fuzzy logic, grid convergence index

## Abstract

A crucial step in any computational fluid dynamics (CFD) analysis is the discretization of the domain because it influences truncation errors, numerical stability, and the convergence of the model. Therefore, the appropriate selection of numerical mesh parameters crucially contributes to the reliability of the obtained results. Therefore, an innovative approach to reducing the mesh-induced error in CFD analysis of an impinging jet using fuzzy logic is proposed within the paper. The flow parameters were obtained using the Reynolds-averaged Navier–Stokes calculations, based on the mesh parameters obtained using the grid convergence index and fuzzy logic, were compared to each other and to experimental research results. The fuzzy logic approach to define mesh parameters turned out to be a very promising method as it allowed us to obtain results that are qualitatively and quantitatively comparable to commonly used but far more time-consuming methods.

## 1. Introduction

An oriented liquid or gaseous impinging jet released against a surface provides an effective and flexible way for transferring large amounts of thermal energy or mass between a given surface and the fluid in many practical applications and developing devices [1,2]. Impinging jets are one of the essential components in many industrial processes, such as surface coating, mixing [3], pollution dispersion, ventilation [4], drying [5], wind engineering [6,7], and direct lift propulsion systems in vertical/short take-off and landing aircraft [8]. The property of enhanced heat transfer between a fluid and an impinged solid target extends the industrial applications of impinging jets to heat treatment; the cooling of materials during forming processes, electronic components [9], turbine components [10,11], combustion engines, and critical machinery structures; and the heating of optical surfaces for defogging [12,13].

Due to their industrial importance and interesting physics, impinging jets have been the object of numerous experimental and theoretical studies concerning the various features of jet dynamics, their interaction with an impinged wall, and the resulting effects on heat and mass transfer [14]. The mechanisms of impinging jets, particularly their near-wall behavior, have been extensively investigated experimentally in studies that are well summarized in Gauntner et al. [15], which focused mainly on basic flow statistics. Another interesting experimental study using laser Doppler velocimetry is presented in Fitzgerald and Garimella [16]. The classification of different parameters that characterize impinging jets and the correlation of the jet Reynolds number, nozzle to wall distance, and turbulent intensity was provided by Tahsini and Mousavi [17]. Other studies were concerned with the heat transfer intensification from impinging jets [8,14]. In Cooper et al. [18], the authors point out that a turbulent jet impinging orthogonally onto a plane surface produces, in the vicinity of the stagnation point, the highest levels of Nusselt number encountered in a single-phase convection. Turbulent structures and heat transfer from an array of impinging jets is presented in Geers et al. [19]. Results of turbulent heat transfer from a slot jet are described in References [20,21].

The flow structure of impinging jets is commonly divided into three main flow regions: the free jet region, stagnation region, and wall jet region [4,22]. The free jet region starts to develop via the entrainment of surrounding fluid in the volume where the fluid jet leaves the nozzle and enters an ambient domain. It is characterized by an initial Kelvin–Helmholtz instability that generates the primary vortex ring structures [4]. The stagnation region exists in the vicinity of the impact area where the normal velocity is suppressed by the pressure–strain mechanism resulting from the presence of an impinged surface and the flow deflects radially. After impinging, the jet develops into a wall jet over the target surface.

Reynolds-averaged Navier–Stokes (RANS), large eddy simulation (LES), direct numerical simulation (DNS), and the lattice Boltzmann method (LBM) have been considered as prospective and valuable tools to provide comprehensive information concerning flow dynamics in both space and time to complement the experimental results concerning impinging jets [2]. Unfortunately, numerical analysis of impinging jets seems to be demanding due to their complex mechanisms and the presence of a stagnation point, high streamline curvature, and the development of a boundary layer at a plain or curved impinged surface [23].

Therefore, the identification of the preferred method for modeling the performance characteristics of an impinging jet, as well as the parameters of the model, are crucial in order to gain an in-depth understanding of this phenomenon and are the aims of this research.

## 2. Materials and Methods

### 2.1. The Research Object

**Figure 1:**
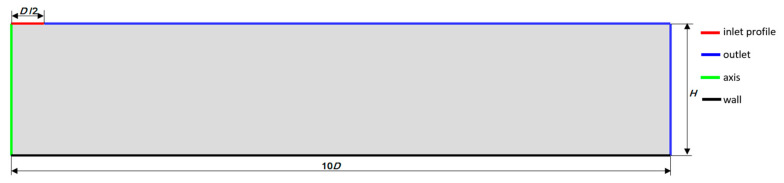
Representation of the research object with boundary conditions (*H*/*D* = 2).

The research object represented a single jet of nozzle diameter equal to *D* impinging perpendicularly on a flat wall. The axial dimension of domain *H* was variable depending on the investigated configuration. The radial dimension of the domain was defined as 10*D*, in accordance with Reference [7,8], in order to reduce the effects of a convective vortex approaching the outlet boundary and to provide realistic exit conditions. The two-dimensional axisymmetric computational domain of the *H/D* ratio equal to 2 with assigned boundary conditions is shown in Figure 1. The boundary conditions assignment was also valid for other *H*/*D* ratios investigated within this research. The inlet boundary condition was defined as profiles of velocity magnitude, turbulent kinetic energy, and turbulent dissipation rate. The profile’s data was obtained from a separate analysis concerning flow in a pipe of diameter *D* and was long enough to produce a fully developed flow at the exit of said pipe. The length of the pipe exceeded the entrance length *L_e_* and was calculated using Equation (1). The Reynolds numbers were defined as *Re* = *V_in_·D*/*ν*, where *V_in_* is a constant inlet velocity to a pipe of diameter *D* and *ν* is the kinematic viscosity.

(1) $$\frac{L_{e}}{D}=4.4Re^{\frac{1}{6}}$$

The profiles were calculated individually for each of the analyzed Reynolds numbers. The obtained exit profiles from the pipe were subsequently applied as inlet profiles to the corresponding impinging jet cases. Such an approach allowed us to minimize the computational domain and simultaneously implement the inlet conditions corresponding to the experimental data obtained for the fully developed flow.

The flowing medium was air of density and viscosity equal to 1.225 (kg/m^3^) and 1.7894 × 10^−5^ (kg/m·s), respectively.

### 2.2. Computational Domain Discretization

The crucial step in any computational fluid dynamics (CFD) analysis is the computational domain discretization (meshing) as it influences the truncation errors, among others [24,25,26]. Moreover, the pre-processing step can influence the numerical stability and convergence of the model [27,28]. These factors crucially contribute to the reliability of the obtained results. The impinging jet is a particularly demanding case in terms of meshing due to the before-mentioned high gradients, streamline curvature, and boundary layer development at the impinged wall. Therefore, special attention must be paid to the numerical mesh, and therefore, the means of mesh-induced error reduction.

The initial mesh was generated using the advanced blocking technique to produce perfectly orthogonal cells. The nodes’ radial spacing and axial spacing were defined in order to easily manipulate the mesh size and investigate its influence on the numerical analysis results. The mesh nodes’ distribution in the radial direction was uniform and the nodes’ radial spacing was defined as *D*/80. However, crucial for the impinging jet case are both the nodes’ distribution and spacing in the axial direction. Therefore, a sophisticated approach was implemented to assure the proper representation of the phenomenon in the direct vicinity of the impinged wall. The axial spacing of the nodes was variable, but the average spacing was equal to *D*/160, which was twice the radial spacing. Moreover, the nodes’ distribution along the axial direction was defined in accordance with the bunching law described using Equation (2):

(2) $$S_{i}=\frac{R-1}{R^{N-1}}\overset{i}{\underset{j=2}{\sum }}R^{j-2}$$

where

*S_i_*: the distance from the node on the impinged wall to node *i*,
*R*: the ratio limited by 0.25 < *R* < 4.0, and
*N*: the total number of nodes resulting from the average axial spacing (*D*/320).

The first layer thickness is the crucial parameter of the bunching law because it highly influences the flow distribution within the said domain. The best practice approach commonly applied in science [7] and industry is to define the first layer thickness by taking into account the value of the dimensionless wall distance *Y^+^* defined by Equation (3). The drawback of this method is the necessity of performing an additional calculation in order to define the flow parameters near the wall, which require calculating *Y^+^*:

(3) $$Y^{+}=\frac{u_{*}y}{\nu }$$

where

*u*: the friction velocity at the nearest wall,
*y*: the distance to the nearest wall, and
*ν*: the local kinematic viscosity of a given fluid.

Moreover, mesh dependency studies need to be performed to assure the independence of the obtained results from the mesh density. The grid convergence index (*GCI*), based on a Richardson extrapolation, is commonly applied and is recommended by the Fluids Engineering Division of the American Society of Mechanical Engineers as a reliable method for mesh dependency analysis. This method requires at least three separate analyses of the same case performed for different mesh resolutions. Therefore, the analysis required is becoming more demanding in terms of time and computational cost.

In order to overcome the above-mentioned disadvantages, an innovative method of defining the first layer thickness is proposed within this paper. The method is based on the fuzzy logic (FL) approach and allows for the definition of a suitable first layer thickness based only on the *H*/*D* ratio and Reynolds number without the necessity of performing the additional time-consuming CFD computations. Both the *GCI* approach and the FL approach for defining mesh parameters are described in detail and compared in the following sections.

### 2.3. Mesh Parameters: Grid Convergence Index Approach

In order to minimize the discretization error and the mesh resolution influence on the RANS analysis results, the *GCI* was calculated for each of the configurations analyzed within the performed research according to the procedure and equations described in References [29,30]. This procedure required CFD results obtained for three meshes of different resolutions to be analyzed; this feature is the greatest inconvenience of the *GCI* method. Therefore, the initial mesh generated according to the procedure described in Section 2.2 was coarsened with the global scaling factor equal to 0.7 and 0.5. Such an approach guaranteed the generation of a proportionally decreased node distribution and spacing in comparison to the initial mesh. Then, the representative mesh size was defined for each of the generated meshes:

(4) $$h={\left[\frac{1}{N}\overset{N}{\underset{i=1}{\sum }}\left(\Delta A_{i}\right)\right]}^{\frac{1}{2}}$$

where

Δ*A_i_*: the area of the *ith* cell, and
*N*: the total number of cells in the computational domain.

The grid refinement factor *r* was calculated using:

(5) $$r=\frac{h_{coarse}}{h_{fine}}$$

Moreover, the following assumption was made:

(6) $$h_{1}<h_{2}<h_{3}$$

The apparent order *p* of the method was solved based on Equation (7) using fixed-point iteration with the initial estimation equal to the first term:

(7) $$p=\frac{\left|ln\left|\frac{\varepsilon_{32}}{\varepsilon_{21}}\right|+ln\left(\frac{r_{21}^{p}-1\cdot sgn\left(\frac{\varepsilon_{32}}{\varepsilon_{21}}\right)}{r_{32}^{p}-1\cdot sgn\left(\frac{\varepsilon_{32}}{\varepsilon_{21}}\right)}\right)\right|}{ln\left(r_{21}\right)}$$

where

(8) $$r_{21}=\frac{h_{2}}{h_{1}},\;\;\;r_{32}=\frac{h_{3}}{h_{2}},\;\;\;\varepsilon_{32}=\phi_{3}-\phi_{2},\;\;\;\varepsilon_{21}=\phi_{2}-\phi_{1}$$

and *ϕ_k_* denotes the value of the variable that is important to the objective of the simulation study for solution obtained with the *kth* mesh. The distance between the impinged wall and the point of the maximal radial velocity divided by the nozzle diameter was selected as the above-mentioned variable.

The extrapolated values were calculated on the basis of Equation (9), approximate relative error was calculated on the basis of Equation (10), and extrapolated relative error was calculated on the basis of Equation (11):

(9) $$\phi_{ext}^{21}=\frac{r_{21}^{p}\phi_{1}-\phi_{2}}{r_{21}^{p}-1},\;\;\;\phi_{ext}^{32}=\frac{r_{32}^{p}\phi_{2}-\phi_{1}}{r_{32}^{p}-1}$$

(10) $$e_{a}^{21}=\left|\frac{\phi_{1}-\phi_{2}}{\phi_{1}}\right|,\;\;\;e_{a}^{32}=\left|\frac{\phi_{2}-\phi_{3}}{\phi_{2}}\right|$$

(11) $$e_{ext}^{21}=\left|\frac{\phi_{ext}^{12}-\phi_{1}}{\phi_{ext}^{12}}\right|,\;\;\;e_{ext}^{32}=\left|\frac{\phi_{ext}^{23}-\phi_{2}}{\phi_{ext}^{23}}\right|$$

Finally, the *GCI* was determined using Equation (12):

(12) $$GCI_{21}=\frac{1.25\cdot e_{a}^{21}}{r_{21}^{p}-1},\;\;\;GCI_{32}=\frac{1.25\cdot e_{a}^{32}}{r_{32}^{p}-1}$$

The convergence type was evaluated using Equation (13):

(13) $$\frac{\varepsilon_{coarse}}{\varepsilon_{fine}}=\left\{ \begin{array}{cc} <0 & \mathrm{oscilatory}\;\mathrm{converged}\\ >1 & \mathrm{not}\;\mathrm{converged}\\ \left[0,1\right] & \mathrm{converged} \end{array}\right.$$

All the values of the above-defined quantities are listed in Table 1.

### 2.4. Mesh Parameters: Fuzzy Logic Approach

Apart from the GCI method described in the previous paragraph, mesh induced error reduction was handled using an innovative approach involving FL, which was developed within the confines of the paper. The preprocessing was carried out according to the procedure described in Section 2.2 with the first layer thickness based on the nozzle diameter and the output from the FL model (*L_f_*). The developed FL model generated the first layer thickness in real-time without any additional CFD analysis like in the case of the GCI method. This capability creates a big advantage of FL over the GCI approach.

The presented approach was undertaken since modeling using the fuzzy logic technique is much simpler, easier, and faster in such cases. FL modeling belongs to the so-called intelligent methods [31,32,33,34]. Since this methodology uses linguistic variables and fuzzy sets, they can deal with imprecise, vague, and uncertain information, as well as provide a qualitative judgment applied to parameters that are quantitative in nature. The QtFuzzyLiteTM fuzzy logic control application (www.fuzzylite.com) was used in this study.

**Figure 2:**
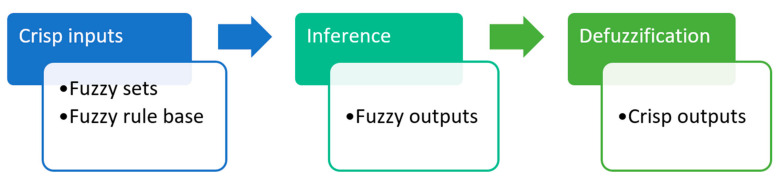
Flow chart of the general fuzzy logic system.

A detailed description of the methodology can be found in Krzywanski and Nowak [35]. The development process of the FL model encompasses modeling inputs and the output as linguistic variables and defining a fuzzy rule base, which describes the behavior of the system [35]. A fuzzifier, fuzzy rule base, inference engine, and defuzzifier constitute the main parts of an FL model. A flowchart of the general fuzzy-logic-based system is depicted in Figure 2.

First, the entire domains of input and output parameters should be covered by fuzzy sets. For the purpose of this work, two inputs (*Re* and *H/D*) as well as one output (*L_f_*) were considered. The output *L_f_* is inversely proportional to the first layer thickness. The inlet nozzle diameter *D* is the proportionality factor according to Equation (14):

(14) $$L_{f}=\frac{D}{S_{1}}$$

where

*L_f_*: output variable,
*D*: nozzle diameter, and
*S_1_*: first layer thickness.

**Figure 3:**
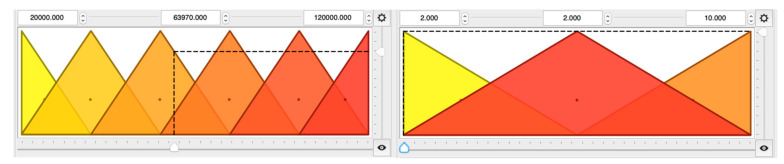
Membership functions for fuzzy sets of the input parameters *Re* (**left**) and *H*/*D* (**right**).

Different strategies for the covering of inputs and outputs using linguistic terms were tested in the study. To make the model more precise, the domain of *Re* was covered using a higher number of linguistic terms (Figure 3 - left). On the other hand, since three different *H*/*D* validation values were available, only three linguistic terms to describe the dependence of *L_f_* on the *H*/*D* (Figure 3 - right) were used. Finally, the inputs *Re* and *H*/*D* are described by six (EL—extremely low, VL—very low, L—low, H—high, VH—very high, EH—extremely high) and three (L—low, M—medium, H—high) triangular, overlapping fuzzy sets, respectively (Figure 3). The use of triangular linguistic variables resulted from the desire to make the model as simple as possible, where this type of variable is one of the most basic in fuzzy logic theory. During the so-called fuzzification stage, a vector of crisp input is transformed into a vector of a membership degree *μ_s_* from the range between 0 and 1, giving a fuzzy set *S*, expressed by the following Zadeh’s notation [31,32,33]:

(15) S= μsi1i1+μsi2i2+…+μsinin

where *i*_1_*, i_2_, …, i_n_* are the input variables.

During the next phase, the fuzzy inference engine generates fuzzy outputs using the so-called IF–THEN fuzzy rule base. In this case, the Takagi–Sugeno inference engine is implemented to determine the fuzzy output variables [34,36]. This approach allows for using polynomial functions, and therefore, a more natural and more precise description of the considered process. The typical rule for input and output *i_n_* and *o*, respectively, can be expressed using the following equation:

(16) $$\mathrm{IF}\;i_{n}\;\mathrm{is}\;S_{n}\;\mathrm{THEN}\;o\;\mathrm{is}\;o=f\left(i_{1},\;i_{2}\right)$$

where *f*(*i_1_*,*i_2_*) is a function of the input parameters.

**Figure 4:**
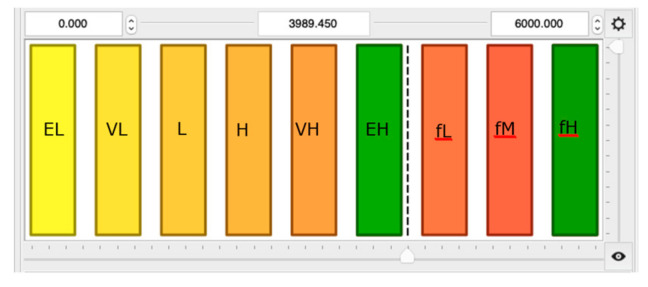
Membership functions for fuzzy sets of the output parameter *L_f_*.

The complete fuzzy rule base for *Re* and *H*/*D* input variables is given in Table 2 and Table 3, respectively, whereas the outputs are shown in Figure 4. Six constant (EL—extremely low, VL—very low, L—low, H—high, VH—very high, EH—extremely high) and three functions (fL, fM, fH) as linguistic terms were used to describe the output variable *L_f_*, where:

(17) $$fL=4.0e-7Re^{2}+0.0972Re-928.3$$

(18) $$fM=3.0e-7Re^{2}+0.0801Re-741.8$$

(19) $$fH=3.0e-7Re^{2}+0.0731Re-725.1$$

Such a dense-defined set of linguistic terms allowed for describing the considered dependencies with a reasonable accuracy. On the other hand, the developed model is simple and useful.

The most computationally efficient methods, i.e., the weighted average method, given by Equation (20), allowed for producing crisp outputs:

(20) $$o=\frac{\sum \mu_{s}\left(\bar{o}\right)\bar{o}}{\mu_{s}\left(\bar{o}\right)}$$

where *o* is the centroid of each membership function.

The approach enabled formalizing an empirical problem using experience rather than knowledge of the theory. The model’s selection parameters were made on the basis of the data obtained from the preliminary CFD analysis *L_f,CFD_* and the calculated *L_f,calc_*, given in Table 4. The maximum relative error *Err* was equal to 20.6%.

**Figure 5:**
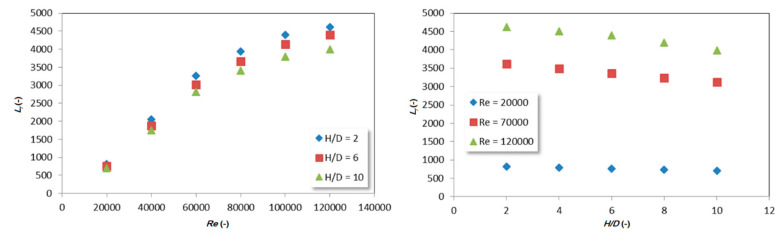
The influence of Reynolds number (**left**) and *H*/*D* ratio (**right**) on the first layer thickness *L_f_*.

Such a developed model allowed for the study of the influence of the input parameters *Re* and *H*/*D* on the *L_f_*, and therefore the first layer thickness. The effect of the Reynolds number of *L_f_* is shown in Figure 5 - left. As the *Re* increased, the *L_f_* also increases, and therefore, the first layer thickness decreased. This was the result of higher velocities contributing to a higher dimensionless wall distance Y^+^ in the direct vicinity of the impinged wall in the case of an increased Reynolds number. On the other hand, the increase in *H*/*D* led to a decrease in *L_f_*, which led to an increase of the first layer thickness (Figure 5 - right). Such behavior can be attributed to lower velocities resulting from the increased dissipation of jet energy in cases of a higher distance from the inlet jet to the impinging wall.

### 2.5. The Computational Model

The numerical research was performed using the Reynolds-averaged Navier–Stokes (RANS) equations solved using the commercial software ANSYS Fluent 19.2, which is based on a finite-volume solution of a mean momentum, energy, and turbulent transport equation. According to the fluent theory guide, the general form of the mass conservation equation for a 2D axisymmetric flow (Equation (21)) is valid for both incompressible and compressible flows. The source *S_m_* is the mass added to the continuous phase from the dispersed second phase (equal to zero in the cases analyzed within this research).

(21) $$\frac{\partial \rho }{\partial t}+\frac{\partial }{\partial x}\left(\rho v_{x}\right)+\frac{\partial }{\partial r}\left(\rho v_{r}\right)+\frac{\rho v_{r}}{r}=S_{m}$$

where

*x*: axial coordinate,
*r*: radial coordinate,
*v_x_*: axial velocity, and
*v_r_*: radial velocity.

The axial (Equation (22)) and radial (Equation (23)) momentum conservation equations are:

(22) $$\begin{array}{l} \frac{\partial }{\partial t}\left(\rho v_{x}\right)+\frac{1}{r}\frac{\partial }{\partial x}\left(r\rho v_{x}v_{x}\right)+\frac{1}{r}\frac{\partial }{\partial r}\left(r\rho v_{r}v_{x}\right)\\ \;=-\frac{\partial p}{\partial x}+\frac{1}{r}\frac{\partial }{\partial x}\left[r\mu \left(2\frac{\partial v_{x}}{\partial x}-\frac{2}{3}\left(\nabla \cdot \vec{v}\right)\right)\right]+\frac{1}{r}\frac{\partial }{\partial r}\left[r\mu \left(\frac{\partial v_{x}}{\partial r}+\frac{\partial v_{r}}{\partial x}\right)\right]+F_{x} \end{array}$$

(23) $$\begin{array}{l} \frac{\partial }{\partial t}\left(\rho v_{r}\right)+\frac{1}{r}\frac{\partial }{\partial x}\left(r\rho v_{x}v_{r}\right)+\frac{1}{r}\frac{\partial }{\partial r}\left(r\rho v_{r}v_{r}\right)\\ \;=-\frac{\partial p}{\partial r}+\frac{1}{r}\frac{\partial }{\partial x}\left[r\mu \left(\frac{\partial v_{r}}{\partial x}+\frac{\partial v_{x}}{\partial r}\right)\right]+\frac{1}{r}\frac{\partial }{\partial r}\left[r\mu \left(2\frac{\partial v_{r}}{\partial r}-\frac{2}{3}\left(\nabla \cdot \vec{v}\right)\right)\right]-2\mu \frac{v_{r}}{r^{2}}\\ \;+\frac{2}{3}\frac{\mu }{r}\left(\nabla \cdot \vec{v}\right)+\rho \frac{v_{z}^{2}}{r}+F_{r} \end{array}$$

where

$\nabla \cdot \vec{v}=\frac{\partial v_{x}}{\partial x}+\frac{\partial v_{r}}{\partial r}+\frac{r}{x}$,
*Μ*: molecular viscosity, and
*v_z_*: swirl velocity.

The selection of a viscous model was based on previous analysis carried out by References [7,18]. The authors point out that k-ε models overpredict the turbulence kinetic energy in the vicinity of the stagnation point, which results in increased entrainment rates of the flow. Therefore, the Reynolds stress model (RSM) with wall reflection effects was chosen as it takes into account multiscale and anisotropic effects [7]. The model constants proposed by Launder [37] were applied: Cmu = 0.09, C1-Epsilon = 1.44, C2-Epsilon = 1.92, C1-PS = 1.8, C2-PS = 0.6, C1’-PS = 0.5, and C2’-PS = 0.3.

An enhanced wall treatment was applied as a near-wall modeling method. According to the fluent theory guide and Kim and Hangan [7], it combines a two-layer model with enhanced wall functions. If the near-wall mesh is fine enough to be able to resolve the laminar sublayer, then the enhanced wall treatment will be identical to the traditional two-layer zonal model.

The coupled scheme of pressure–velocity coupling and the quadratic upstream interpolation for convective kinematics (QUICK) scheme for turbulent kinetic energy, turbulent dissipation rate, and Reynolds stresses were applied.

The solution control parameters were the explicit relaxation factors of momentum (0.3) and pressure (0.3), as well as the under-relaxation factors of turbulent kinetic energy (0.8), turbulent dissipation rate (0.8), turbulent viscosity (1), and Reynolds stresses (0.5).

The model convergence was defined on the basis of qualitative and quantitative monitoring of the standard residuals, as well as the mass flow rate imbalance between inlet and outlets.

### 2.6. The Experimental Research

The results of the numerical modeling were compared to the experimental data available from the ERCOFTAC Classic Database, test case No. 25: Normally Impinging Jet from a Circular Nozzle. The data was originally obtained during detailed laboratory experiments described in Cooper et al. [18]. The experiments provided measurements of a turbulent jet impinging orthogonally onto a large plain wall. Before the discharge, the air passed along a smooth pipe sufficiently long to generate fully developed flow at the exit of the nozzle. A hot-wire technique was applied in the measurements. Two Reynolds numbers were considered, 2.3 × 10^4^ and 7.0 × 10^4^, and the spacing between the wall and the nozzle ranged from two to ten nozzle diameters with particular emphasis on two and six. The available experimental data concerned the mean velocity profile in the vicinity of the wall and the three Reynolds stress components. The investigated configurations of the experimental research are listed in Table 5.

Absolute accuracies of the experimental data are as follows [18]: the maximum mean velocity was accurate within 2%, estimated uncertainty of root-mean-square fluctuating velocities were 4% (*u*’) and 6% (*v*’), and turbulent shear–stress (*uv*) uncertainties were of the order of 9%.

## 3. Results and Discussion

The results of the RANS numerical modeling with the application of the RSM viscous model and enhanced wall treatment are presented for the configurations listed in Table 5 (different *H*/*D* ratios and jet flow Reynolds numbers). The presented CFD results were obtained for numerical meshes considered as converged in terms of the GCI method and meshes of parameters generated with the FL approach. The CFD results were benchmarked against the detailed experimental results obtained within research described in Cooper et al. [18].

**Figure 6:**
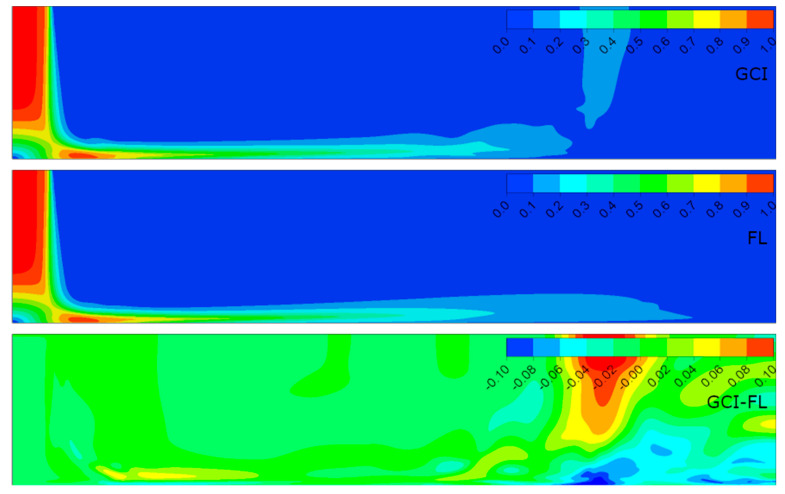
Normalized velocities for configuration #A (*Re* = 2.3 × 10^4^, *H*/*D* = 2) obtained for meshes parametrized using GCI and FL methods.

**Figure 7:**
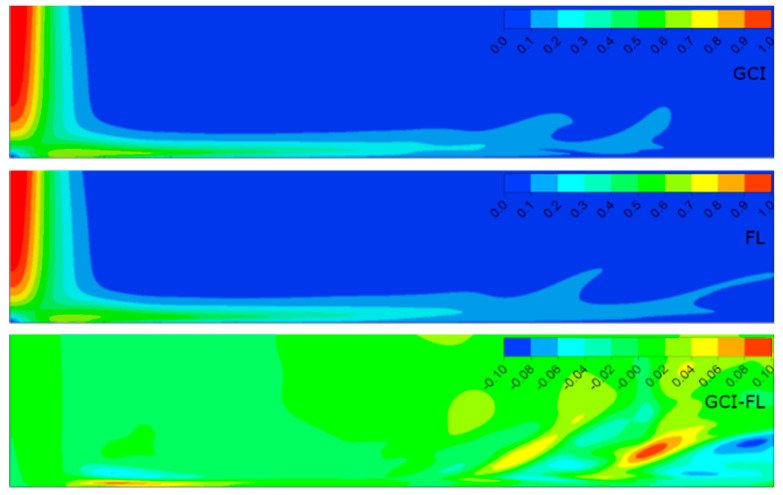
Normalized velocities for configuration #B (*Re* = 2.3 × 10^4^, *H*/*D* = 6) obtained for meshes parametrized using GCI and FL methods.

**Figure 8:**
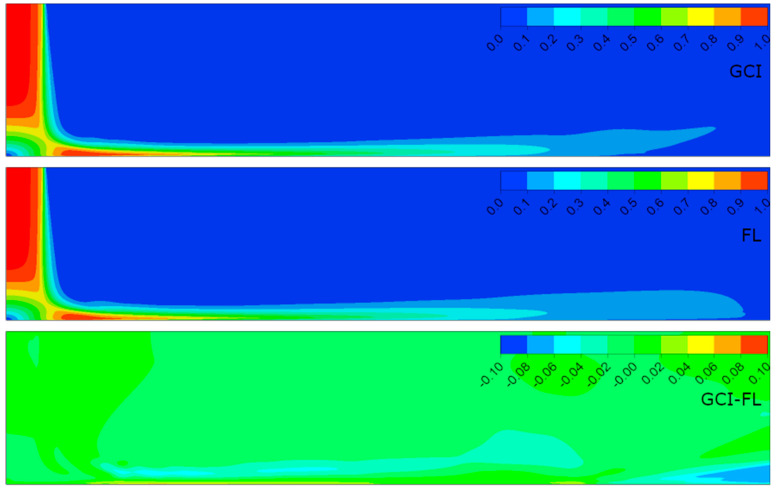
Normalized velocities for configuration #C (*Re* = 7.0 × 10^4^, *H*/*D* = 2) obtained for meshes parametrized using GCI and FL methods.

**Figure 9:**
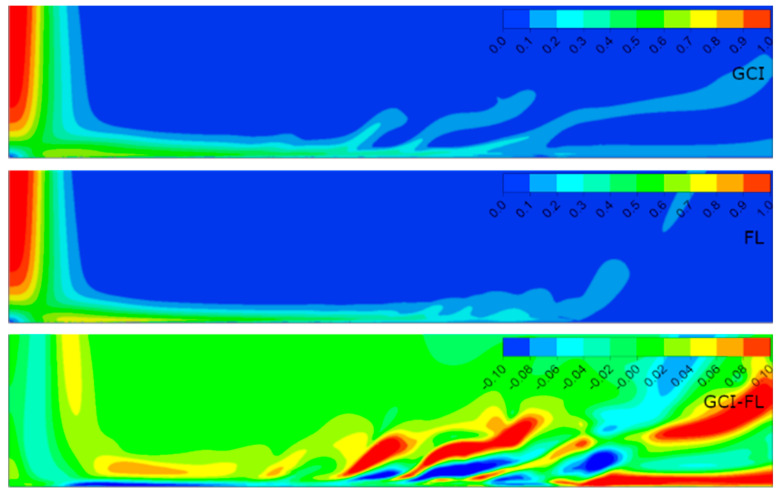
Normalized velocities for configuration #D (*Re* = 7.0 × 10^4^, *H*/*D* = 6) obtained for meshes parametrized using GCI and FL methods.

Figure 6, Figure 7, Figure 8 and Figure 9 present the normalized velocities with the average jet velocity at the nozzle exit at the area of the computational domain extending 2*D* axially and 10*D* radially for all the analyzed configurations. The flow field was obtained for the meshes parametrized with both the GCI and FL methods. Moreover, the difference between them for straightforward comparison is depicted.

It is noticeable that the flow field in all configurations consisted of three distinguished structures: the free jet, impinging, and wall jet regions. The free jet length depended on the jet-to-wall distance resulting from the *H*/*D* ratio. The impinging point was directly beneath the jet nozzle center with the highest pressure, and at the same time, the lowest velocity, creating a stagnation point characterized by a high-pressure gradient and flow deformation. Due to the high pressure, the fluid accelerated parallel to the surface. The velocity gradient created a shear stress which led to a high heat transfer according to the Reynolds analogy between shear and heat transfer [12]. The flow gradually lost its kinetic energy along the path from the stagnation point and transformed into the wall jet. A similar behavior of flow was observed in References [5,23,38].

The results concerning the normalized velocities (Figure 6, Figure 7, Figure 8 and Figure 9) were obtained for meshes generated using the GCI method (upper contour plot), results for meshes generated using the FL method (middle contour plot), and the difference plots (GCI-FL) for straightforward comparison (bottom contour plot). The difference plots were calculated for each data point (node) as follows: the normalized velocity obtained using the GCI method minus the normalized velocity obtained using the FL method. The presented span of the normalized velocity difference for all the configurations was −0.1 to 0.1 in order to make the comparison between individual cases more precise. Therefore, the blue regions in the difference plots indicate the areas of higher normalized velocity obtained using the FL method, red regions indicate the areas of higher normalized velocity obtained using the GCI method, and green regions indicate similar values of normalized velocities obtained using both methods. Thus, it can be stated that the results were qualitatively and quantitatively similar, although they were not identical. Configuration #C was characterized by the lowest differences as the prevailing color was green. On the other hand, the most significant differences were obtained for configuration #D as there were relatively large regions of red color indicating higher values of normalized velocity from the GCI method compared to the FL method. It must be emphasized that the differences mostly occurred in the area away from the stagnation point and impinged wall; therefore, it can be assumed that they were induced not by the mesh parameters, but may have resulted from convergence issues that occurred randomly in the model. Quantitative differences between the analyzed configurations are presented in Table 6 in the form of minimal and maximal values of normalized velocities in the difference plots (GCI-FL).

Not significant but noticeable dissimilarities also occurred in the thickness of the film in the impinging region, especially near the stagnation point. The film thickness depended on both the Reynolds number and the *H*/*D* ratio according to the data in Table 7. The table presents the distance between the impinged wall and the point of maximal radial velocity normalized to the nozzle diameter (*ϕ*) for each of the analyzed configurations. In the case of configurations with the same *H*/*D* ratio, the film was thicker for lower Reynolds numbers: configuration #A compared to #C and configuration #B compared to #D. In the case of configurations of the same Reynolds number, the film was thicker for higher *H*/*D* ratios: configuration #B compared to #A and configuration #D compared to #C. Therefore, it can be concluded that the film thickness was inversely proportional to the Reynolds number and proportional to the *H*/*D* ratio.

The velocity of the fluid decreased with distance from the stagnation point and hence the film became thicker. The most important changes in the film thickness occur within a 5*D*–9*D* radial distance from the impinging point. Similar flow behavior was also observed by Hosain [12]. Moreover, the separation of the wall jet flow could be noticed mostly in the case of configurations #B and #D (*H*/*D* = 6). This may have resulted from the lower turbulence kinetic energy of the jet close to the impinged wall caused by the three times higher axial dimension of the computational domain.

**Figure 10:**
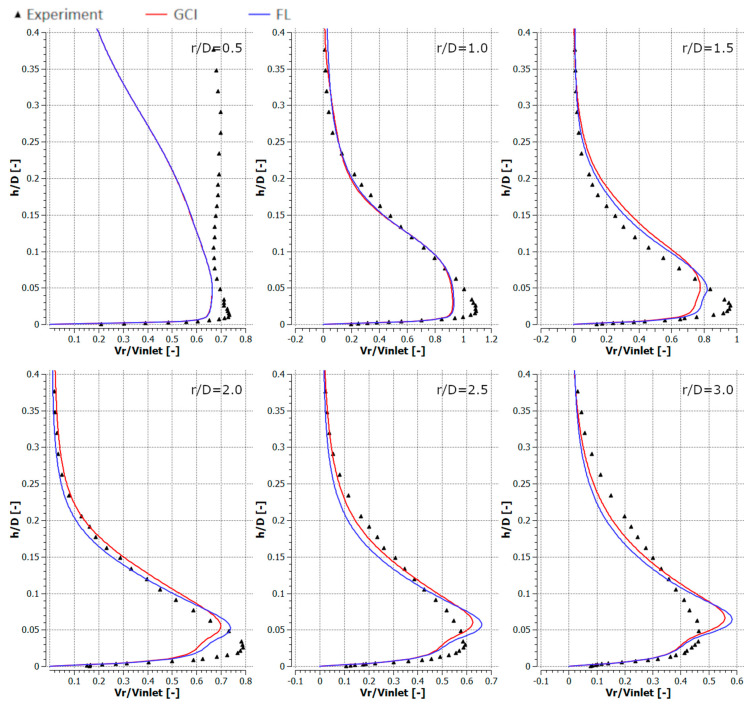
Normalized radial velocities for configuration #A (*Re* = 2.3 × 10^4^, *H*/*D* = 2).

**Figure 11:**
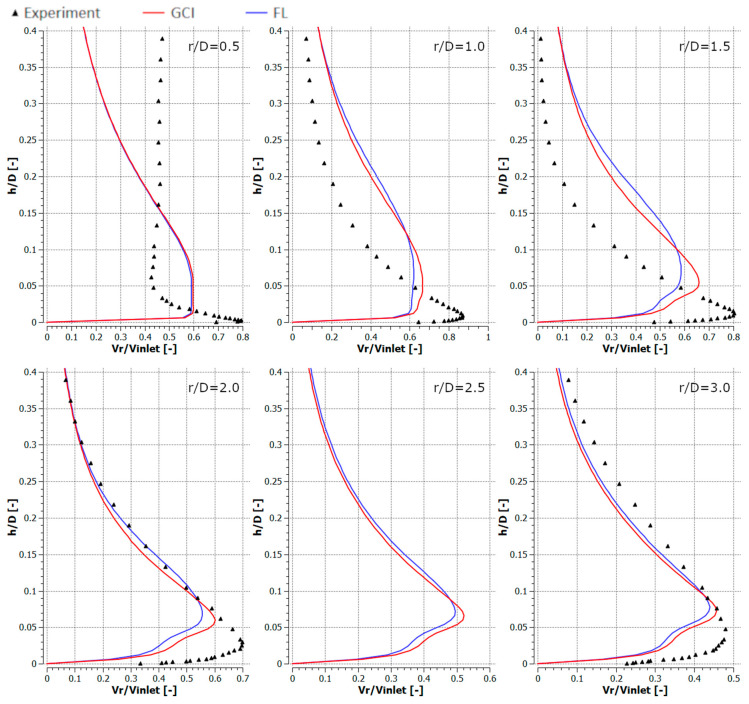
Normalized radial velocities for configuration #B (*Re* = 2.3 × 10^4^, *H*/*D* = 6).

**Figure 12:**
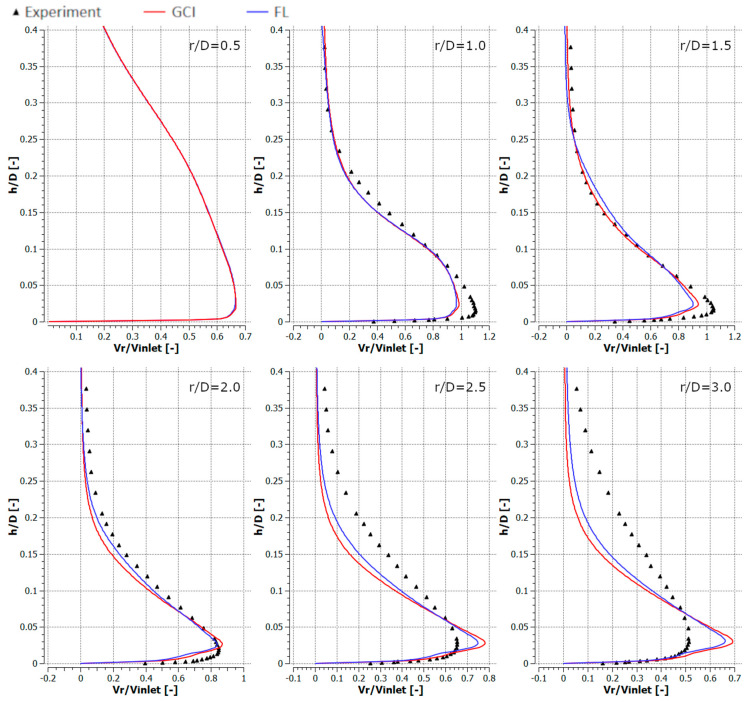
Normalized radial velocities for configuration #C (*Re* = 7.0 × 10^4^, *H*/*D* = 2).

**Figure 13:**
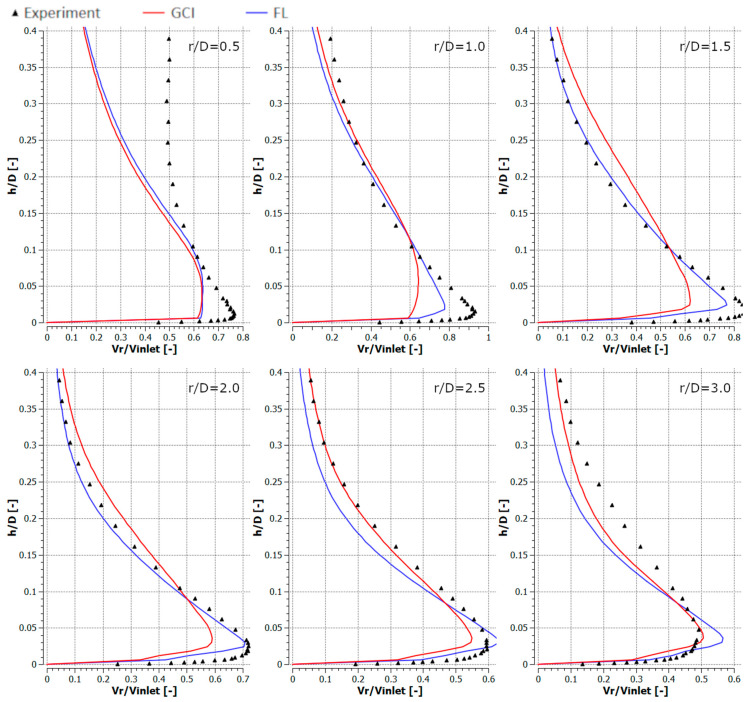
Normalized radial velocities for configuration #D (*Re* = 7.0 × 10^4^, *H*/*D* = 6).

Figure 10, Figure 11, Figure 12 and Figure 13 depict the radial component of the velocity normalized with the average jet velocity at the nozzle exit for configurations #A, #B, #C, and #D. The presented curves were also obtained for the meshes parametrized using GCI (red lines) and FL (blue lines) for *r*/*D* equal to 0.5, 1.0, 1.5, 2.0, 2.5, and 3.0. The numerical modeling results were compared with experimental data (black triangles). All analyzed configurations confirmed a good qualitative conformity of the numerical results obtained with meshes generated using the GCI and FL approaches.

**Figure 14:**
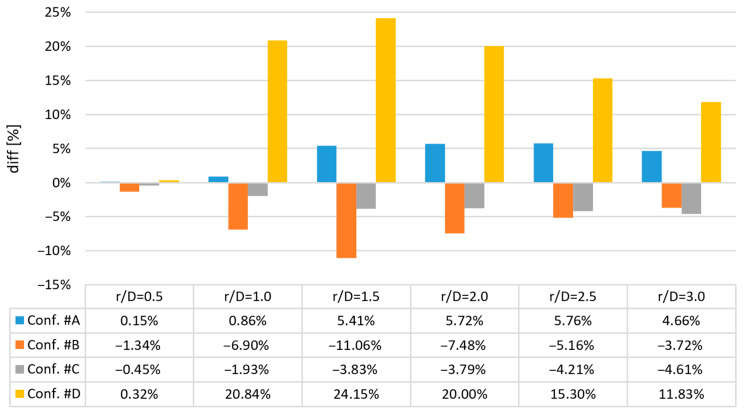
Quantitative differences between the results concerning the maximal radial velocity obtained using the GCI method and the FL method.

The crucial parameter for the jet impingement is the maximal radial velocity; therefore, the quantitative differences between the results concerning the maximal radial velocity obtained with the GCI method and the FL method are presented in Figure 14 and were calculated according to Equation (24):

(24) diff=maxVrVinletFL−maxVrVinletGCImaxVrVinletGCI×100%

where

*V_r_:* radial component of the velocity (m/s), and
*V*_inlet_: average jet velocity at the inlet (m/s).

In the case of configurations #A and #C, the results were also quantitatively similar, especially for *r*/*D* equal to 0.5 and 1.0. For other values of *r*/*D*, the differences did not exceed 5.76% of the normalized radial velocity. The maximal differences in the normalized radial velocity obtained using CFD for different meshes were at the level of 11.06% in the case of configuration #B and 24.15% in the case of configuration #D.

The results obtained using numerical modeling were in good qualitative agreement with the experimental results except for the case of *r/D* = 0.5 for all the analyzed configurations. The difference for *r/D* = 0.5 may have resulted from the fact that the measurements were carried out with a TSI IFA-100 two-channel hot-wire anemometer equipped with standard DISA single-wire probes. Therefore, the influence of the axial velocity component on the measurement results was significantly nearer the axis of the inlet jet. The CFD underestimated the maximal radial velocities for *r/D* less than 2.0 and overestimated it for higher *r/D* in the cases of configurations #A (Figure 10) and #C (Figure 12). Numerical results concerning radial velocity for configuration #D (Figure 13) behaved similarly as for configurations #A and #C, except for *r/D* = 2.5, in which the CFD results obtained for GCI were lower and for FL were higher than the experimental results. The normalized radial velocity obtained experimentally was higher in comparison to CFD for all *r/D* values in the case of configuration #B. The vertical location of the point of maximal radial velocity was situated higher for the CFD results (all configurations and all *r/D*) in comparison to the experimental data.

## 4. Conclusions

Impinging jets provide an effective way of transferring large amounts of mass between an impinged wall and a fluid.

The separate CFD flow simulation in the inlet nozzle (in order to generate the inlet profile), allowed for the minimization of the computational domain and simultaneously implemented inlet conditions corresponding to the experimental data obtained for a fully developed flow.

The impinging jet is a particularly demanding case in terms of meshing due to the complicated physics taking place mainly near the impinged wall. Therefore, special attention must be paid to reduce the mesh induced error in CFD analysis.

The thickness of the first layer of the numerical mesh is the crucial parameter influencing the flow distribution in numerical analysis concerning impinging jets. For that reason, it was defined using two approaches: the commonly used grid convergence index (GCI) and the fuzzy logic (FL) approach. The pre-computed data obtained from the CFD analysis was used to develop the FL model within the confines of the research. Once the FL model was developed, it was used to define the first layer thickness for cases concerning a wide range of Reynolds numbers or *H*/*D* ratios without the necessity to perform time-demanding CFD pre-analysis aiming to define the appropriate first layer thickness. The developed FL model generated the first layer thickness in real-time, and this capability created a big advantage for the FL approach over the GCI approach. The main disadvantage of the FL model is that its application is limited to the impinging jet cases.

The flow patterns for various Reynolds numbers and *H*/*D* ratios obtained using RANS calculations with the application of the RSM viscous model and enhanced wall treatment based on the mesh parameters obtained with GCI and FL were qualitatively and quantitatively comparable within an error not exceeding 5.76%, 11.06%, 4.61%, and 24.15% depending on the analyzed case. The thickness of the film in the impinging region near the stagnation point depended on the Reynolds number and the *H*/*D* ratio. The most important changes in the modeled film thickness occurred within a 5*D*-9*D* radial distance from the impinging point.

The results concerning normalized radial velocities obtained using numerical modeling were qualitatively comparable to experimental results but the quantitative differences (calculated similarly as for the GCI and FL comparison) were up to 26.1%, 28.6%, 35.4%, and 30.9% depending on the analyzed configuration.

The vertical location of the point of maximal radial velocity was situated further from the impinged wall for the CFD results in comparison to the experimental data.

The fuzzy logic approach for defining the optimal mesh parameters in CFD analysis of impinging jets turned out to be a very promising method of discretization error reduction as it allowed for obtaining reliable results in a definitely less demanding way in terms of time and computational cost when compared to the commonly used grid convergence index method.

## Figures and Tables

**Table 1:** Values calculated using Equations (4)–(13).

*N* (-)	*ϕ* (-)	*h* (-)	*r* (-)	*ε* (-)	*ε_coarse_/ε_fine_*(-)	*p* (-)	*ϕ_ext_* (-)	*e_a_* (%)	*e_ext_* (%)	*GCI* (%)
512000	0.0425	0.0014	1.429	−0.0040	0.0228 converged	10.02	0.0426	9.29%	0.27%	0.34%
250880	0.0386	0.0020	1.400	−0.0001			0.0386	0.23%	0.01%	0.01%
128000	0.0385	0.0028	-	-			-	-	-	-

**Table 2:** Fuzzy rule base for *Re*.

*L_f_*	EL	VL	L	H	VH	EH
*Re*	EL	VL	L	H	VH	EH

**Table 3:** Fuzzy rule base for *H*/*D*.

*L_f_*	fL	fM	fH
*H/D*	L	M	H

**Table 4:** The FL data.

*Re*	*H/D*	*L_f,exp_*	*L_f,calc_*	*Err* (%)
23000	2	1000	1015.6	1.56
70000	6	1000	938.6	6.14
23000	2	3000	3619.1	20.6
70000	6	3000	3358.9	11.96

**Table 5:** Experimentally investigated configurations of the research object.

	Reynolds Number *Re* (-)	Height to Diameter Ratio *H*/*D* (-)
**Configuration #A**	2.3 × 10^4^	2
**Configuration #B**	2.3 × 10^4^	6
**Configuration #C**	7.0 × 10^4^	2
**Configuration #D**	7.0 × 10^4^	6

**Table 6:** Quantitative comparison of the minimal and maximal differences of normalized velocities.

	Minimal Normalized Velocity for GCI-FL (-)	Maximal Normalized Velocity for GCI-FL (-)
**Configuration #A**	−0.131	0.121
**Configuration #B**	−0.087	0.095
**Configuration #C**	−0.077	0.065
**Configuration #D**	−0.187	0.182

**Table 7:** The influence of the Reynolds number and *H*/*D* ratio on the distance between the impinged wall and the point of maximal radial velocity normalized to the nozzle diameter (*ϕ*).

Configuration	*ϕ* (-)
**Configuration #A**	0.04250
**Configuration #B**	0.05284
**Configuration #C**	0.03323
**Configuration #D**	0.04603
